# Association between triglyceride glucose index and atherosclerotic plaques and Burden: findings from a community-based study

**DOI:** 10.1186/s12933-022-01638-x

**Published:** 2022-10-11

**Authors:** Mengxing Wang, Lerong Mei, Aoming Jin, Xueli Cai, Jing Jing, Suying Wang, Xia Meng, Shan Li, Tiemin Wei, Yongjun Wang, Yuesong Pan

**Affiliations:** 1 Department of Neurology, Beijing Tiantan Hospital, Capital Medical University, No.119, South 4th Ring West Road, Fengtai District, 100070 Beijing, China; 2 China National Clinical Research Center for Neurological Diseases, Beijing, China; 3 Cerebrovascular Research Lab, Lishui Hospital, Zhejiang University School of Medicine, Lishui, China; 4 Department of Neurology, Lishui Hospital, Zhejiang University School of Medicine, Lishui, China; 5 Department of Cardiology, Lishui Hospital, Zhejiang University School of Medicine, Lishui, China

**Keywords:** Insulin resistance, Triglyceride glucose index, HOMA-IR, Atherosclerosis

## Abstract

**Background:**

Insulin resistance is an important cause of cardiovascular events and cerebral infarction development. We aimed to investigate the association of the triglyceride glucose (TyG) index with atherosclerotic burden and plaques in coronary, intra- and extracranial arteries in participants with non-diabetes, and compared the results with that of the homeostasis model assessment of insulin resistance (HOMA-IR).

**Methods:**

Participants without diabetes in the PolyvasculaR Evaluation for Cognitive Impairment and vaScular Events (PRECISE) study were included. We categorized participants by tertiles of the TyG index and the concordance/discordance of the TyG index and HOMA-IR. Discordance was defined as a TyG index equal to or greater than the median and HOMA-IR less than the median, or vice versa. The atherosclerosis plaques and burden in coronary, intra- and extracranial arteries were evaluated. The association of HOMA-IR and TyG index with the presence of atherosclerotic plaques and atherosclerotic burden was assessed by binary and ordinal logistic regression models, respectively.

**Results:**

Among 2,719 included participants, the average age was 60.9 (± 6.6) years, and 53.0% were female. Both TyG index and HOMA-IR were associated with increased odds of coronary/intra- and extracranial atherosclerotic plaques and burden after adjustment for age, sex, currenting smoking and drinking (all P < 0.05). However, the association between HOMA-IR and intracranial atherosclerosis was not statistically significant after adjustment for all potential confounders. Discordantly high TyG index with HOMA-IR had a higher odd of extracranial plaque (odds ratio [OR]: 1.34, 95% confidence interval [CI]: 1.04–1.71), extracranial atherosclerotic burden (common odds ratio [cOR]: 1.35, 95% CI 1.06–1.71), coronary plaque (OR: 1.30, 95% CI 1.01–1.68) and segment stenosis score (cOR: 1.39, 95% CI 1.09–1.78) as compared with concordantly low TyG index with HOMA-IR. The TyG index had a better net reclassification improvement ability than HOMA-IR for atherosclerotic plaques when adding to baseline model.

**Conclusion:**

Elevated TyG index was associated with increased odds of atherosclerosis in coronary/intra- and extracranial arteries. Compared with HOMA-IR, the TyG index was more strongly associated with intracranial atherosclerosis. Moreover, discordantly high TyG index with HOMA-IR was also important for atherosclerosis identification.

**Supplementary Information:**

The online version contains supplementary material available at 10.1186/s12933-022-01638-x.

## Background

Insulin resistance, as a risk factor for metabolic diseases, was involved in the development of cardiovascular disease [1] and associated with increased stroke risk [2]. Previous studies have reported an association between insulin resistance and early arteriosclerotic lesions [3], the development of which is the main pathological cause of cardiovascular artery disease and stroke [4, 5]. Among biomarkers of insulin resistance, the homeostasis model assessment of insulin resistance (HOMA-IR) index is the most common method for measuring insulin resistance [6]. Recently, the triglyceride glucose (TyG) index, a noninsulin-based and inexpensive approach index to evaluate insulin resistance, has been developed. The TyG index is calculated by fasting glucose and triglyceride, and proposed as a useful surrogate marker of insulin resistance [7]. Some studies have shown that the TyG index had a better value for assessing insulin resistance [8–10] and was more closely related to coronary artery stenosis and calcification than HOMA-IR [8, 9]. However, few studies have focused on comparison of relationship of insulin resistance indexes with comprehensive evaluation of atherosclerotic burden and plaques, especially in intra- and extracranial arteries.

We therefore aimed to investigate the association of the TyG index with comprehensive atherosclerotic burden and plaques in coronary, intra- and extracranial arteries in nondiabetic community-dwelling adults and compared the results with that of HOMA-IR.

## Methods

### Participants

The PolyvasculaR Evaluation for Cognitive Impairment and vaScular Events (PRECISE) study recruited 3067 participants aged 50 to 75 from a cluster sample of 6 villages and 4 communities in Lishui city between May 2017 and September 2019. Details of the study have been published elsewhere[11]. Participants with contraindications to computed tomography angiography (CTA) and magnetic resonance imaging (MRI), mental illness, and life expectancy of less than 4 years were excluded. All participants were comprehensively evaluated for stenosis and plaques in coronary, intra- and extracranial arteries with advanced vascular imaging techniques. The Ethics Committee approved the study protocol of Beijing Tiantan Hospital (IRB Approval No. Ky2017-010-01) and Lishui Hospital (IRB Approval No. 2016-42), and all participants have signed informed consents before enrollment. Participants with self-reported diabetes previously diagnosed by a physician or using of glucose-lowering or insulin medications were excluded from this analysis. Non-diabetic participants who underwent CTA examination, MRI examination, and blood sampling were included in the analysis.

### Anthropometric, clinical and socio‑demographic parameters

All anthropometric, clinical and socio-demographic data were collected face to face by trained interviewers following the standard protocols, which included age, sex, education level, body mass index (BMI), smoking, drinking, relevant medical history, and self-reported medication use. BMI was calculated as weighted (kg) divided by the square of height (m^2^) [6]. Hypertension was defined as hypertension previously diagnosed by physicians or current use of antihypertensive agents or systolic blood pressure ≥ 140 mmHg or diastolic blood pressure ≥ 90 mmHg [12]. History of dyslipidemia was determined by self-reported dyslipidemia previously diagnosed by a physician usually according to Chinese guidelines for the prevention and treatment of adult dyslipidemia[13] or current use of lipid-lowering drugs (e.g., statins, fibrates).

### Biological measurements

Venous blood samples were collected in the morning after fasting for at least 8 h at night and transported to Tiantan Hospital biobank for preservation by the cold chain. Biological measurements, including fasting glucose, fasting insulin and triglyceride, were validated and measured in the Central Laboratory of Beijing Tiantan Hospital under strict quality control standards. Insulin resistance was assessed by using HOMA-IR model and the TyG index. The TyG index was determined as ln (fasting triglycerides [mg/dL] × fasting glucose [mg/dL]/2)] [8] and the HOMA-IR was calculated as (fasting insulin [µU/ mL] × fasting glucose [mmol/L])/22.5 [6]. Participants were categorized into 4 concordance/discordance groups: high TyG index/ high HOMA-IR, high TyG index/ low HOMA-IR, low TyG index/ high HOMA-IR, low TyG index/ low HOMA-IR [14]. A TyG index equal to or greater than the median and less than the median were defined as high TyG index and low TyG index, respectively, and similar definition was applied to HOMA-IR. The high TyG index /low HOMA-IR and high HOMA-IR/ low TyG index groups were considered as discordance.

### Measurements of coronary atherosclerosis

The CTA was performed on a dual-source CT scanner (SOMATOM Force, Siemens Healthineers, Forchheim, Germany) to examine coronary atherosclerosis burden. The presence of coronary plaque was defined as a structure > 1mm^2^ within and/or adjacent to the coronary artery lumen, which could be clearly distinguished from the vessel lumen and the surrounding pericardial/myocardial tissue [15]. The evaluated coronary artery segments included the left main trunk, anterior descending branch, diagonal branch, circumflex branch, obtuse margin branch, middle branch, right coronary artery, posterior descending branch and posterior lateral branch. Coronary stenosis was quantified by visual estimation, and each artery segments graded as following: score 0, minimal (< 30%); score 1, mild (30–49%); score 2, moderate (50–69%); score 3, severe (> 70%) [16]. The segment stenosis score (SSS) was calculated as a measure of the overall coronary artery plaque burden, and was graded and summed for each coronary segment as none to severe plaque (0–3) based on the extent of obstruction (total score ranging: 0 to 27) [15]. The SSS was further categorized into three grades (grade 0, 0 score; grade 1, 1–3 scores; grade 2, ≥ 4 scores). We calculated the Cohens κ to evaluated interobserver agreement for the presence of plaque and artery stenosis (Cohen κ = 0.96 and 0.92).

### Measurements of intra- and extracranial atherosclerosis

The 3.0T scanner (Ingenia 3.0T, Philips, Best, The Netherlands) was used to detect intra-/extra-cranial atherosclerotic plaques and stenosis. The imaging sequences included three-dimensional time-of-flight magnetic resonance angiography (3D-TOF-MRA) and three-dimensional isotropic high-resolution black-blood T1w vessel wall imaging (3D T1w VWI). The presence of intra- and extracranial atherosclerosis plaques was defined as eccentric wall thickening with or without luminal stenosis identified on the 3D T1w VWI images in comparison to the corresponding location on the 3D TOF MR angiogram at MR angiography [17, 18]. The evaluated intracranial arterial segments included the bilateral internal carotid, anterior cerebral arteries (A1, A2), the middle cerebral artery (M1, M2), posterior cerebral arteries (P1, P2), and basal vertebral arteries (V4). The extracranial arterial segments included the common carotid, proximal internal carotid artery, and vertebral artery (V1, V2, V3) [17]. The Warfarin-Aspirin Symptomatic Intracranial Disease Trial (WASIDT) [19] was used to assess the luminal stenosis in the intracranial arteries, and the North American Symptomatic Carotid Endarterectomy Trial (NASCET) [20] was used to measure the luminal stenosis in the extracranial arteries. According to the degree of atherosclerotic plaques and stenosis, semi-quantitative score was used to evaluate atherosclerotic conditions (0, no atherosclerotic plaque; 1 atherosclerotic plaque without evident lumen stenosis or stenosis < 50%; 2, stenosis 50-69%; 3, stenosis 70-99%; 4, occlusion) [21]. The intracranial atherosclerotic burden was calculated through summing the intracranial atherosclerotic scores of intracranial arterial segments, which were categorized into four grades of 0, 1, 2–3, and ≥ 4 scores [21]. Similarly, the extracranial atherosclerotic burden was counted through extracranial arterial segments, and categorized into four grades of 0, 1, 2–3, and ≥ 4 scores[21]. We calculated the Cohens κ to assess the interrater reliability for the presence of plaques and artery stenosis ≥ 50% in intra- and extracranial artery (Cohen κ = 0.97 and 0.79 for intracranial artery and Cohen κ = 0.94 and 0.86 for extracranial artery).

### Statistical methods

The characteristics of the participants were displayed according to the tertiles of insulin resistance index. Data are presented as mean ± standard deviation (SD) or median [interquartile range (IQR)] for continuous variables and as the frequency (%) for categorical variables. We used ANOVA or Kruskal-Wallis test for continuous variables and χ2 test or Fisher’s exact for categorical variables. The association of TyG index or HOMA-IR with the presence of atherosclerotic plaques was analyzed by binary logistic regressions and with the atherosclerotic burden by ordinal logistic regression models. The odds ratio (OR) or common odds ratio (cOR) with 95% confidence intervals (CI) were estimated. Logistic regression models were also applied to estimate odds for atherosclerosis associated with concordance/discordance groups. We used 2 models: model 1, unadjusted; model 2, adjusted for age, sex, current smoking, current drinking, BMI, estimated glomerular filtration rate, low-density lipoprotein levels, medical history of transient ischemic attack, stroke, coronary artery disease, hypertension, dyslipidemia, and use of antihypertensive and antiplatelet medications. Restricted cubic splines with 5 knots were constructed to model a non-linear relationship between HOMA-IR or TyG and the presence of atherosclerotic plaques. The area under the receiver operating characteristic curve (AUC), the integrated discrimination improvement (IDI), and the net reclassification improvement (NRI) were determined to assess the predictive performance of insulin resistance index [7].

Two-tailed P value < 0.05 was considered statistically significant, and all analyses were performed using SAS version 9.4 (SAS Institute Inc, Cary, NC).

## Results

### Baseline characteristics

**Fig. 1:**
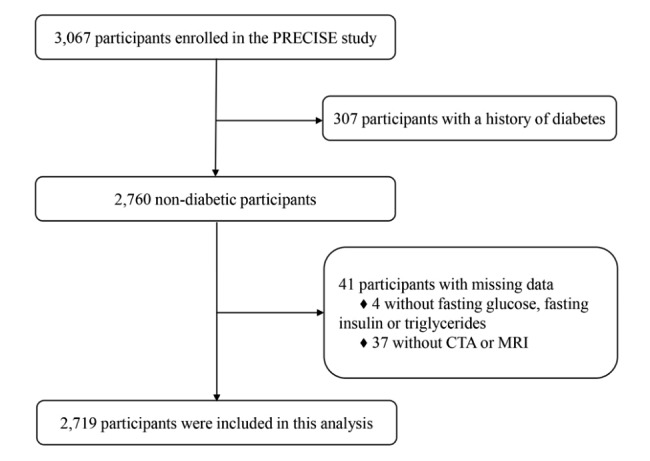
**Flowchart of the included participants.** PRECISE, PolyvasculaR Evaluation for Cognitive Impairment and vaScular Events; CTA, computed tomography angiography; MRI, magnetic resonance imaging

Among the 3,067 enrolled participants, 307 participants with diabetes, 4 with missing fasting glucose, fasting insulin or triglyceride, and 37 without CTA or MRI examinations were excluded, and a total of 2,719 participants were available for final analysis (Fig. 1). The demographic and clinical characteristics of the included participants are shown according to the tertiles of TyG index in Table 1. Among the included participants, the average age was 60.9 (± 6.6) years, and 53.0% were women. Participants with higher TyG had more frequent dyslipidemia and hypertension, and higher levels of BMI, fasting glucose, fasting insulin, and triglyceride (P < 0.05). Baseline characteristics compared across the 4 concordance/discordance groups and different HOMA-IR tertiles are shown in Additional file 1: Table S1-S2.

**Table 1:** Baseline characteristics according to TyG groups

Characteristics	Total n = 2719	Tertile1* n = 906	Tertile2 n = 907	Tertile3 n = 906	P value
Age(years)	60.9 ± 6.6	61.3 ± 6.7	60.9 ± 6.6	60.5 ± 6.4	0.04
Female (n, %)	1441(53.0)	465(51.3)	498(54.9)	478(52.8)	0.31
Current smoking, n (%)	566(20.8)	184(20.3)	193(21.3)	189(20.9)	0.88
Current drinking, n (%)	522(19.2)	170(18.8)	171(18.9)	181(20.0)	0.77
History of comorbidities (n, %)					
Hypertension	1095(40.3)	270(29.8)	364(40.1)	461(50.9)	< 0.001
Dyslipidemia	533(19.6)	77(8.5)	174(19.2)	282(31.3)	< 0.001
TIA	9(0.3)	2(0.2)	6(0.7)	1(0.1)	0.10
Stroke	64(2.4)	24(2.7)	19(2.1)	21(2.3)	0.74
Coronary artery disease	10(0.4)	4(0.4)	3(0.3)	3(0.3)	0.90
Medication history (n, %)					
Lipid-lowering medicine	90(3.3)	16(1.8)	38(4.2)	36(4.0)	< 0.001
Statins	82(3.0)	16(1.8)	35(3.9)	31(3.4)	0.02
Fibrate	5(0.2)	0(0.0)	2(0.2)	3(0.3)	0.23
Other	4(0.2)	0(0.0)	1(0.1)	3(0.3)	0.17
Antihypertensive medications	667(24.5)	152(16.8)	211(23.3)	304(33.6)	< 0.001
BMI (km/m^2^)	23.7 ± 2.0	22.4 ± 2.9	23.9 ± 2.9	24.7 ± 2.8	< 0.001
FPG (mmol/L)	5.66 ± 0.98	5.4 ± 0.5	5.6 ± 0.7	6.0 ± 1.4	< 0.001
Fasting insulin (mmol/L)	6.2(4.4–8.8)	4.8(3.4–6.5)	6.2(4.6–8.7)	8.1(5.9–10.9)	< 0.001
TG (mg/ dL)	1.7 ± 1.2	0.9 ± 0.2	1.5 ± 0.2	2.9 ± 1.4	< 0.001
eGFR (mL/min/1.73 m^2^)	102.3 ± 11.7	103.2 ± 10.8	101.6 ± 12.3	102.1 ± 12.0	0.02
LDL (mmol/L)	2.8 ± 0.8	2.6 ± 0.7	3.0 ± 0.8	2.8 ± 0.9	< 0.001

TyG, triglyceride glucose index; TIA, transient ischemia attack; BMI, body mass index; FPG, fasting plasma glucose; TG, triglyceride; eGFR, estimated glomerular filtration rate; SD, standard deviation

* Tertiles of the TyG index, < 8.53; 8.53-9.00; >9.00

### Association of insulin resistance and atherosclerosis

**Fig. 2:**
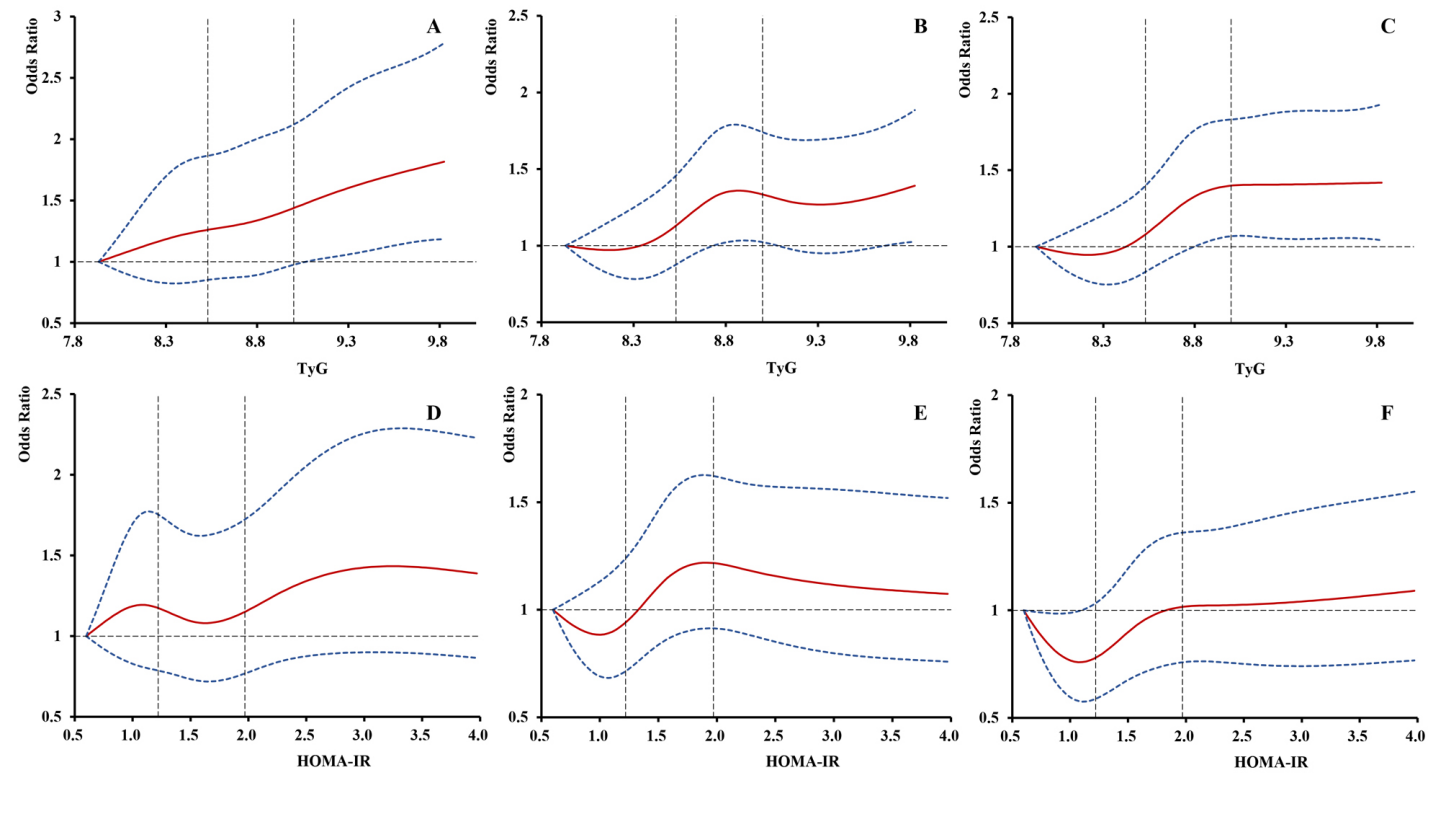
**Association of insulin resistance with the presence of coronary, intracranial and extracranial arteries plaque.** Multivariable-adjusted ORs for the presence of intracranial(A), extracranial (B) and coronary (C) plaques based on restricted cubic spines with 5 knots (5th, 25th, 50th, 75th, and 95th percentiles) of TyG index. Multivariable-adjusted ORs for the presence of intracranial(D), extracranial (E) and coronary (F) plaques based on restricted cubic spines with 5 knots (5th, 25th, 50th, 75th, and 95th percentiles) of HOMA-IR. HOMA-IR, homeostasis model assessment insulin resistance; TyG, the triglyceride glucose index All models adjusted for age, sex, current smoking, current drinking, BMI, estimated glomerular filtration rate, low-density lipoprotein levels, medical history of TIA, stroke, coronary disease, hypertension and dyslipidemia, using of antihypertensive and antiplatelet medications

Of the 2,719 nondiabetic participants, 432(15.89%) had intracranial plaques, 975(35.86%) had extracranial plaques and 1160(42.66%) had coronary plaques. Compared with the first tertile, the third tertile of TyG was associated with increased odds of the presence of intracranial plaques (OR,1.45; 95%CI, 1.09–1.92, P = 0.01), intracranial atherosclerotic burden (cOR,1.40; 95%CI, 1.06–1.85, P = 0.02), the presence of extracranial plaques (OR,1.25; 95%CI, 1.01–1.55, P = 0.04), extracranial atherosclerotic burden (cOR: 1.28, 95% CI, 1.04–1.58, P = 0.02), the presence of coronary plaques (OR: 1.42, 95% CI, 1.14–1.76, P = 0.002) and segment stenosis score (cOR: 1.40, 95% CI, 1.14–1.73, P = 0.002) after adjusting for potential covariates (Table 2). Similar results were observed for the HOMA-IR in model1, but the association between HOMA-IR and the presence of intra- and extracranial atherosclerotic plaques, and intracranial atherosclerotic burden was not significant after adjustment for all confounders (all P > 0.05). In Fig. 2, multivariable-adjusted spline regression models showed a positive relationship between TyG index or HOMA-IR and the presence of coronary, intra- and extracranial plaques.

**Table 2:** Association of the TyG index/HOMA-IR with atherosclerosis in coronary, intra- and extracranial arteries

Atherosclerotic plaque	The TyG index						HOMA-IR				
	n/N (%)	Model1* OR/cOR(95%CI)	P	Model2† OR/cOR(95%CI)	P		n/N (%)	Model1* OR/cOR(95%CI)	P	Model2† OR/cOR(95%CI)	P
Presence of intracranial plaque											
T1	113/906(12.5)	Reference		Reference			128/906(14.1)	Reference		Reference	
T2	148/907(16.3)	1.42(1.09–1.85)	0.01	1.27(0.96–1.68)	0.10		136/904(15.0)	1.16(0.89–1.52)	0.28	0.96(0.72–1.27)	0.75
T3	171/906(18.9)	1.75(1.34–2.27)	< 0.001	1.45(1.09–1.92)	0.01		168/909(18.5)	1.49(1.15–1.94)	0.003	1.06(0.78–1.45)	0.69
Pre 1-SD	-	1.26(1.14–1.40)	< 0.001	1.19(1.06–1.33)	0.003		-	1.13(1.03–1.24)	0.01	1.01(0.91–1.13)	0.82
P for trend	-		< 0.001		0.01		-		0.002		0.58
Intracranial atherosclerotic burden‡											
T1	-	Reference		Reference			-	Reference		Reference	
T2	-	1.39(1.06–1.81)	0.02	1.22(0.92–1.61)	0.16		-	1.16(0.89–1.52)	0.27	0.95(0.71–1.26)	0.70
T3	-	1.73(1.33–2.24)	< 0.001	1.40(1.06–1.85)	0.02		-	1.49(1.15–1.93)	0.003	1.05(0.78–1.43)	0.74
Pre 1-SD	-	1.25(1.13–1.39)	< 0.001	1.16(1.04–1.30)	0.009		-	1.12(1.02–1.22)	0.014	0.999(0.89–1.12)	0.98
P for trend	-		< 0.001		0.02		-		0.002		0.61
Presence of extracranial plaque											
T1	285/906(31.5)	Reference		Reference			315/906(34.8)	Reference		Reference	
T2	353/907(38.9)	1.43(1.18–1.74)	< 0.001	1.30(1.06–1.60)	0.01		329/904(36.4)	1.23(1.01–1.51)	0.04	1.17(0.95–1.45)	0.14
T3	337/906(37.2)	1.33(1.09–1.62)	0.005	1.25(1.01–1.55)	0.04		331/909(36.4)	1.28(1.05–1.56)	0.02	1.24(0.98–1.57)	0.07
Pre 1-SD	-	1.13(1.04–1.22)	0.003	1.12(1.02–1.22)	0.01		-	1.03(0.95–1.11)	0.49	1.01(0.92–1.10)	0.90
P for trend	-		0.007		0.052		-		0.03		0.09
Extracranial atherosclerotic burden§											
T1	-	Reference		Reference			-	Reference		Reference	
T2	-	1.43(1.18–1.73)	< 0.001	1.31(1.07–1.60)	0.008		-	1.27(1.04–1.54)	0.02	1.22(0.99–1.49)	0.06
T3	-	1.34(1.11–1.63)	0.003	1.28(1.04–1.58)	0.02		-	1.29(1.06–1.57)	0.01	1.27(1.01–1.60)	0.04
Pre 1-SD	-	1.14(1.05–1.23)	0.001	1.13(1.04–1.23)	0.004		-	1.03(0.96–1.11)	0.44	1.01(0.93–1.10)	0.80
P for trend	-		0.004		0.03		-		0.02		0.06
Presence of coronary plaque											
T1	328/906(36.2)	Reference		Reference			356/906(39.3)	Reference		Reference	
T2	414/907(45.6)	1.63(1.33–1.99)	< 0.001	1.40(1.13–1.72)	0.002		378/904(41.8)	1.36(1.11–1.67)	0.003	1.16(0.94–1.44)	0.17
T3	418/906(46.1)	1.72(1.41–2.09)	< 0.001	1.42(1.14–1.76)	0.002		426/909(46.9)	1.75(1.43–2.15)	< 0.001	1.33(1.05–1.69)	0.02
Pre 1-SD	-	1.22(1.12–1.32)	< 0.001	1.14(1.04–1.24)	0.002		-	1.25(1.13–1.37)	< 0.001	1.10(0.99–1.21)	0.08
P for trend	-		< 0.001		0.002		-		< 0.001		0.02
Segment stenosis score||											
T1	-	Reference		Reference			-	Reference		Reference	
T2	-	1.36(1.12–1.66)	0.002	1.23(1.004–1.52)	0.046		-	1.34(1.10–1.64)	0.004	1.21(0.98–1.49)	0.08
T3	-	1.62(1.33–1.97)	< 0.001	1.40(1.14–1.73)	0.002		-	1.62(1.33–1.98)	< 0.001	1.30(1.03–1.65)	0.03
Pre 1-SD	-	1.20(1.11–1.30)	< 0.001	1.14(1.05–1.24)	0.002		-	1.20(1.11–1.31)	< 0.001	1.11(1.02–1.21)	0.01
P for trend	-		< 0.001		0.002		-		< 0.001		0.04

ORs were estimated for the presence of intracranial, extracranial and coronary plaque; cORs were used for intra-/extra-cranial atherosclerotic burden and segment stenosis score

HOMA-IR, homeostasis model assessment insulin resistance; TyG, the triglyceride glucose index

*Model 1: adjusted for age, sex, current smoking and current drinking;

†Model 2: adjusted for age, sex, current smoking, current drinking, BMI, estimated glomerular filtration rate, low-density lipoprotein levels, medical history of TIA, stroke, coronary disease, hypertension and dyslipidemia, using of antihypertensive and antiplatelet medications

‡ The intracranial atherosclerosis burden was graded according to the sum of intracranial atherosclerosis scores, including 0, 1, 2–3 and ≥ 4 scores

§ The extracranial atherosclerosis burden was graded according to the sum of extracranial atherosclerosis scores, including 0, 1, 2–3 and ≥ 4 scores

|| Segment stenosis score, the total number of segments with any plaque was graded: 0, 1–3, and ≥ 4 scores

**Fig. 3:**
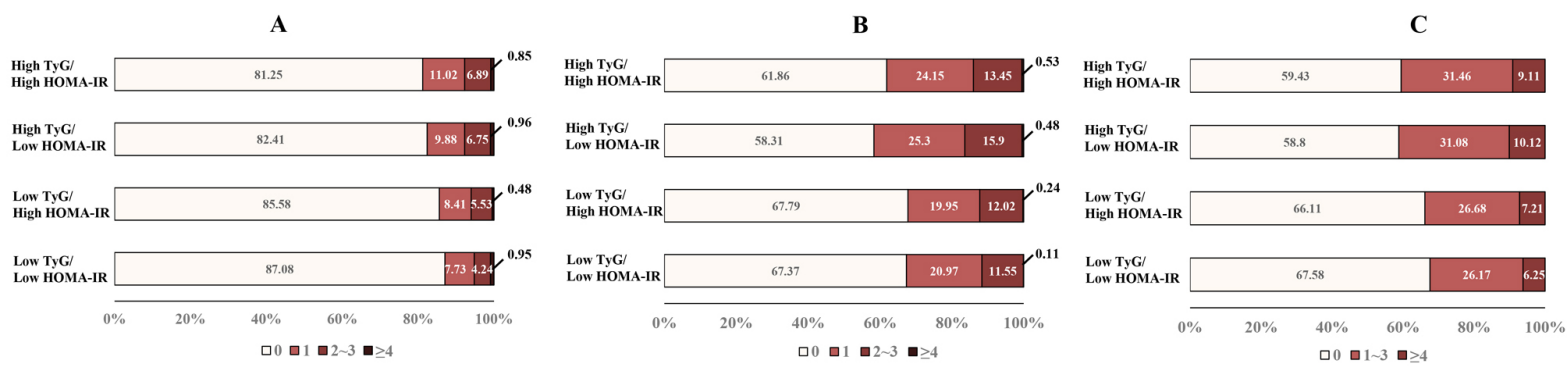
**Bar charts of atherosclerotic burden of concordance/discordance groups** Distribution of intracranial atherosclerotic burden(A), extracranial atherosclerotic burden(B), and Segment stenosis score(C) in accordance and discordance groups HOMA-IR, homeostasis model assessment insulin resistance; TyG, the triglyceride glucose index The intracranial atherosclerosis burden was graded according to the sum of intracranial atherosclerosis scores, including 0, 1, 2–3 and ≥ 4 scores The extracranial atherosclerosis burden was graded according to the sum of extracranial atherosclerosis scores, including 0, 1, 2–3 and ≥ 4 scores Segment stenosis score, the total number of segments with any plaque was graded: 0, 1–3, and ≥ 4 scores

Figure 3 shows the different proportions of participants with coronary, intra- and extracranial atherosclerotic burden in 4 concordance/discordance groups. In the concordance/discordance analysis (Table 3), discordantly high TyG index with HOMA-IR had a greater odd of extracranial plaque (OR: 1.34, 95% CI 1.04–1.71), extracranial atherosclerotic burden (cOR: 1.35, 95% CI 1.06–1.71), coronary plaque (OR: 1.30, 95% CI 1.01–1.68) and segment stenosis score (cOR: 1.39, 95% CI 1.09–1.78) as compared with concordantly low TyG index with HOMA-IR. For the outcome of the presence of atherosclerotic plaques, we found the TyG index had a better net reclassification improvement ability than HOMA-IR when adding to baseline models (NRI, 9.57 -18.01%, all P < 0.05) (Additional file 1: Table S3).

**Table 3:** Association of the concordance/discordance groups with atherosclerosis in coronary, intra- and extracranial arteries

Atherosclerotic plaque	TyG /HOMA-IR	n/N (%)	Model1*		Model2†	
			OR/cOR(95%CI)	P	OR/cOR(95%CI)	P
Presence of intracranial plaque	Low TyG /Low HOMA-IR	122/944(12.92)	Reference		Reference	
	Low TyG /High HOMA-IR	60/415(14.42)	1.2(0.85–1.68)	0.30	0.98(0.68–1.42)	0.93
	High TyG /Low HOMA-IR	73/416(17.59)	1.45(1.05-2.00)	0.02	1.31(0.94–1.82)	0.11
	High TyG /High HOMA-IR	177/944(18.75)	1.66(1.29–2.15)	< 0.001	1.28(0.95–1.71)	0.10
Intracranial atherosclerotic burden‡	Low TyG /Low HOMA-IR	-	Reference		Reference	
	Low TyG /High HOMA-IR	-	1.19(0.85–1.67)	0.31	0.99(0.69–1.42)	0.96
	High TyG /Low HOMA-IR	-	1.47(1.07–2.02)	0.02	1.31(0.94–1.82)	0.11
	High TyG /High HOMA-IR	-	1.66(1.28–2.14)	< 0.001	1.26(0.94–1.68)	0.12
Presence of extracranial plaque	Low TyG /Low HOMA-IR	308/944(32.63)	Reference		Reference	
	Low TyG /High HOMA-IR	134/415(32.21)	1.11(0.87–1.43)	0.40	1.10(0.85–1.44)	0.47
	High TyG /Low HOMA-IR	173/416(41.69)	1.39(1.09–1.77)	0.007	1.34(1.04–1.71)	0.02
	High TyG /High HOMA-IR	360/944(38.14)	1.39(1.14–1.68)	< 0.001	1.35(1.08–1.68)	< 0.001
Extracranial atherosclerotic burden§	Low TyG /Low HOMA-IR	-	Reference		Reference	
	Low TyG /High HOMA-IR	-	1.15(0.90–1.47)	0.28	1.14(0.88–1.48)	0.33
	High TyG /Low HOMA-IR	-	1.40(1.10–1.76)	0.005	1.35(1.06–1.71)	0.02
	High TyG /High HOMA-IR	-	1.40(1.16–1.69)	< 0.001	1.38(1.11–1.71)	< 0.001
Presence of coronary plaque	Low TyG /Low HOMA-IR	354/944(37.50)	Reference		Reference	
	Low TyG /High HOMA-IR	164/415(39.42)	1.29(1.001–1.66)	0.049	1.08(0.82–1.42)	0.58
	High TyG /Low HOMA-IR	195/416(46.99)	1.46(1.14–1.86)	0.003	1.30(1.01–1.68)	0.04
	High TyG /High HOMA-IR	447/944(47.35)	1.78(1.46–2.16)	< 0.001	1.40(1.12–1.75)	< 0.001
Segment stenosis score||	Low TyG /Low HOMA-IR	-	Reference		Reference	
	Low TyG /High HOMA-IR	-	1.25(0.97–1.61)	0.08	1.11(0.85–1.45)	0.44
	High TyG /Low HOMA-IR	-	1.47(1.16–1.88)	0.001	1.39(1.09–1.78)	0.009
	High TyG /High HOMA-IR	-	1.65(1.36-2.00)	< 0.001	1.39(1.11–1.73)	0.004

ORs were estimated for the presence of intracranial, extracranial and coronary plaque; cORs were used for Intra-/extra-cranial atherosclerotic burden and segment stenosis score

HOMA-IR, homeostasis model assessment insulin resistance; TyG, the triglyceride glucose index

*Model 1: adjusted for age, sex, current smoking and current drinking;

†Model 2: adjusted for age, sex, current smoking, current drinking, BMI, estimated glomerular filtration rate, low-density lipoprotein levels, medical history of transient ischemia attack, stroke, coronary disease, hypertension and dyslipidemia, using of antihypertensive and antiplatelet medications

‡ The intracranial atherosclerosis burden was graded according to the sum of intracranial atherosclerosis scores, including 0, 1, 2–3 and ≥ 4 scores

§ The extracranial atherosclerosis burden was graded according to the sum of extracranial atherosclerosis scores, including 0, 1, 2–3 and ≥ 4 scores

|| Segment stenosis score, the total number of segments with any plaque was graded: 0, 1–3, and ≥ 4 scores

The TyG index and HOMA-IR were divided into high and low groups by using median as cut-off value. Median TyG: 8.76; median HOMA-IR: 1.55

## Discussion

This cross-sectional study showed significant associations between the TyG index and intra- and extracranial, and coronary atherosclerosis after adjustment for confounders. The TyG index was more strongly associated with increased odds for the presence of intracranial atherosclerotic plaques and burden than HOMA-IR after adjustment for potential covariates. Moreover, the TyG index for atherosclerotic plaques had a better improvement ability than HOMA-IR, and the concordance/discordance analysis also reveal extra clinical value of the TyG index.

Several studies suggested that TyG index was recognized as a risk factor for cardiovascular disease [1, 22, 23]. Ki-Bum Won et al. [22] demonstrated the TyG index was associated with an increased risk of severe coronary artery disease in patients with near-normal renal function, and Kahui Park et al. [1] showed elevated TyG index level was significantly associated with the progression of coronary artery calcification regardless of other conventional risk factors. In addition, The TyG index was more independently associated with coronary artery atherosclerosis than HOMA-IR in healthy Korean adults [9]. Similarly, the Asan Medical Center [24] demonstrated the TyG index rather than HOMA-IR had an independent association with the presence of coronary artery disease in non-diabetic patients. Although there was a difference in outcome measures, our study arrived at a similar conclusion that the TyG index but not HOMA-IR was strongly associated with coronary atherosclerosis from the perspective of the presence of coronary atherosclerosis plaques and burden.

Moreover, previous studies have also reported the association between insulin resistance and intra- and extra-cranial arterial disease [25, 26]. There was very little literature linking insulin resistance with extracranial atherosclerosis, but some literature showed that insulin resistance could cause intracranial atherosclerosis by affecting arterial dilation [26, 27]. The Kongcun Town Study [28] demonstrated that the insulin resistance was related to asymptomatic intracranial arterial stenosis. Similarly, the AsIA cohort study [29] suggested insulin resistance and related metabolic abnormalities emerged as an important mechanism involved in the development of intracranial atherosclerotic disease. On the contrary, based on Transcranial Doppler Ultrasonography examination, the APAC study [25] showed elevated TyG index was associated with a significantly higher risk of extracranial artery stenosis, but not with intracranial artery stenosis. Compared with previous studies, our study used a high-resolution black-blood T1w vessel wall imaging technique to evaluate intra- and external stenosis, and provided additional evidence for significant correlation between the TyG index and intra- and external burden and plaques. Meanwhile, we further demonstrated that the TyG index had a better value in identifying intra- and external atherosclerosis plaques than HOMA-IR.

Although the mechanisms of TyG index had superior association than HOMA-IR with atherosclerosis have not yet been fully elucidated, two possible explanations have been proposed. First, the two insulin resistance indexes may reflect different aspects of insulin resistance. The TyG index was thought to reflect the insulin resistance of both the hepatic and peripheral tissues[9], whereas HOMA-IR only reflects insulin resistance of hepatic tissues [10]. The peripheral insulin resistance was a useful marker of atherosclerosis risk [30]. Thus, the TyG index showed a significantly association with atherosclerosis. Second, one important mechanism between insulin resistance and atherosclerosis might be hyperglycemic damage [22]. Besides, when triglyceride levels are very high, the triglyceride-rich chyle particles are too large to cross the endothelial barrier into the intima, and then also cause atherosclerosis [31]. The TyG index takes into account the effects of fasting glucose and triglyceride, and mirrors the physiological condition of glucose and lipid at the same time [32]. Evaluated by two common clinical indicators (fasting glucose and triglyceride), the TyG index also have a potential clinical advantage that it was noninsulin-based and less costly than HOMA-IR [8]. This may suggest that the TyG index could be a more appropriate indicator for identifying high-risk atherosclerosis patients in clinical practice.

### Strengths and limitations

Our study has several strengths. First, with advanced high-resolution imaging techniques, our study comprehensively assessed the intra-/extra-cranial atherosclerotic plaques and stenosis, and calculated atherosclerosis burden. Second, the concordance/discordance analysis helped us further understood the extra clinical value of the TyG index via the disagreements between the TyG index and HOMA-IR.

Some potential limitations also require careful consideration in the present study. First, the cross-sectional data limits the exploration of causality. The longitudinal study of atherosclerosis may better indicate the association between TyG index and progression of atherosclerosis diseases. Second, this study only includes the Chinese population and restricts the generalization of the results. Third, our study population comes from the same neighborhoods, and the inherent selection bias may influence our results to some extent. Despite these limitations, this study provides evidence for the correlation between TyG index and coronary, intracranial or extracranial atherosclerosis. These results need to be further validated in other large-scale populations.

## Conclusion

Elevated TyG index was associated with increased odds of atherosclerosis in coronary, intra- and extracranial arteries. Compared with HOMA-IR, the TyG index was more strongly associated with intracranial atherosclerosis. Besides, the TyG index could be a more appropriate predictor to identify high-risk atherosclerosis patients.

## Electronic supplementary material

Below is the link to the electronic supplementary material.

Supplementary Material 1

## Data Availability

The datasets generated and analyzed during the current study are available from the corresponding author on reasonable request.

## Abbreviations

TyG index: the triglyceride glucose index
HOMA-IR: the homeostasis model assessment of insulin resistance
the PRECISE study: the PolyvasculaR Evaluation for Cognitive Impairment and vaScular Events study
OR: odds ratio
CI: confidence interval
cOR: common odds ratio
CTA: contraindications to computed tomography angiography
MRI: magnetic resonance imaging
BMI: body mass index
WASIDT: the Warfarin-Aspirin Symptomatic Intracranial Disease Trial
NASCET: the North American Symptomatic Carotid Endarterectomy Trial
IQR: interquartile range
AUC: the receiver operating characteristic curve
IDI: the integrated discrimination improvement
NRI: the net reclassification improvement
