# Supplementary material for: Association between triglyceride glucose index and atherosclerotic plaques and Burden: findings from a community-based study
Source: Cardiovasc Diabetol. 2022 Oct 11;21:204. doi: 10.1186/s12933-022-01638-x (PMC9555111; doi:10.1186/s12933-022-01638-x)
Supplement: Supplementary file 1 — Supplementary Material 1 [file 12933_2022_1638_MOESM1_ESM.docx]

**Additional file 1**

**♦ Table S1.** Baseline characteristics according to HOMA-IR groups.

**♦ Table S2.** Baseline Characteristics of the concordance/discordance groups.

**♦ Table S3.** Prediction performance of the TyG index and HOMA-IR.

**Table S1. Baseline characteristics according to HOMA-IR groups.**

| Characteristics | Total  n=2719 | Tertile1*  n=906 | Tertile2  n=904 | Tertile3  n=909 | P value |
| --- | --- | --- | --- | --- | --- |
| Age(years) | 60.9±6.6 | 61.4±6.7 | 60.6±6.4 | 60.7±6.6 | 0.04 |
| Female (n, %) | 1441(53.0) | 353(39.0) | 518(57.3) | 570(62.7) | <0.01 |
| Current smoking, n (%) | 566(20.8) | 286(31.6) | 154(17.0) | 126(13.9) | <0.01 |
| Current drinking, n (%) | 522(19.2) | 255(28.2) | 152(16.8) | 115(12.6) | <0.01 |
| History of medical (n, %) |  |  |  |  |  |
| Hypertension | 1095(40.3) | 241(26.6) | 366(40.5) | 488(53.7) | <0.01 |
| Dyslipidemia | 533(19.6) | 93(10.3) | 191(21.1) | 249(27.4) | <0.01 |
| TIA | 9(0.3) | 2(0.2) | 6(0.66) | 1(0.11) | 0.09 |
| Stroke | 64(2.4) | 22(2.4) | 17(1.7) | 25(2.8) | 0.47 |
| Coronary disease | 10(0.37) | 4(0.44) | 2(0.22) | 4(0.44) | 0.67 |
| Medication history (n, %) |  |  |  |  |  |
| lipid-lowering medicine | 90(3.3) | 16(1.8) | 27(3.0) | 47(5.2) | <0.01 |
| Statins | 82(3.0) | 15(1.7) | 26(2.9) | 41(4.5) | 0.002 |
| Fibrate | 5(0.2) | 1(0.1) | 1(0.1) | 3(0.3) | 0.45 |
| Other | 4(0.2) | 0(0.0) | 0(0.0) | 4(0.4) | 0.02 |
| Antihypertensive medicine | 667(24.5) | 118(13.0) | 218(24.1) | 331(36.4) | <0.01 |
| BMI (km/m^2^) | 23.7±2.0 | 21.9±2.4 | 23.7±2.6 | 25.4±2.9 | <0.01 |
| FPG (mmol/L) | 5.7±1.0 | 5.3±0.6 | 5.6±0.6 | 6.1±1.4 | <0.01 |
| Fasting insulin (mmol/L) | 6.2(4.4-8.8) | 3.8(3.0-4.5) | 6.2(5.6-7.1) | 10.3(8.8-12.7) | <0.01 |
| TG (mg/ dL) | 1.7±1.2 | 1.3±0.8 | 1.8±1.2 | 2.2±1.3 | <0.01 |
| eGFR (mL/min/1.73 m^2^) | 102.3±11.7 | 102.8±11.4 | 102.2±11.4 | 101.8±12.4 | 0.22 |
| LDL (mmol/L) | 2.8±0.8 | 2.7±0.7 | 2.9±0.8 | 2.8±0.8 | <0.01 |

HOMA-IR, homeostasis model assessment insulin resistance; TIA, transient cerebral ischemia; BMI, body mass index; FPG, fasting plasma glucose; TG, Triglyceride; eGFR, estimated glomerular filtration rate; SD, standard deviation.

* Tertiles of the HOMA-IR, <1.22; 1.22-1.97; >1.97.

**Table S2. Baseline** **characteristics of the concordance/discordance groups**.

| Characteristics | Total  n=2719 | Low TyG/ Low HOMA-IR  n=944 | Low TyG/ High HOMA-IR  n=416 | High TyG/Low HOMA-IR  n=415 | High TyG/High HOMA-IR  n=944 | P value |
| --- | --- | --- | --- | --- | --- | --- |
| Age(years) | 60.9±6.6 | 61.2±6.8 | 60.7±6.7 | 61.2±6.4 | 60.6±6.4 | 0.13 |
| Female (n, %) | 1441(53.0) | 453(48.0) | 281(67.6) | 152(36.6) | 555(58.8) | <0.01 |
| Current smoking, n (%) | 566(20.8) | 233(24.7) | 43(10.3) | 132(31.8) | 158(16.7) | <0.01 |
| Current drinking, n (%) | 522(19.2) | 203(21.5) | 47(11.3) | 129(31.1) | 143(15.2) | <0.01 |
| History of comorbidities (n, %) |  |  |  |  |  |  |
| Hypertension | 1095(40.3) | 260(27.5) | 174(41.8) | 169(40.7) | 492(52.12) | <0.01 |
| Dyslipidemia | 533(19.6) | 85(9.0) | 63(15.1) | 97(23.4) | 288(30.5) | <0.01 |
| TIA | 9(0.3) | 5(0.5) | 2(0.5) | 1(0.2) | 1(0.1) | 0.40 |
| Stroke | 64(2.4) | 24(2.5) | 11(2.6) | 7(1.7) | 22(2.3) | 0.78 |
| Coronary disease | 10(0.4) | 5(0.5) | 1(0.2) | 1(0.2) | 3(0.3) | 0.78 |
| Medication history (n, %) |  |  |  |  |  |  |
| lipid-lowering medicine | 90(3.3) | 16(1.7) | 18(4.3) | 10(2.4) | 46(4.9) | <0.01 |
| Statins | 82(3.0) | 16(1.7) | 18(4.3) | 9(2.2) | 39(4.1) | <0.01 |
| Fibrate | 5(0.2) | 0(0.0) | 0(0.0) | 1(0.2) | 4(0.4) | 0.14 |
| Other | 4(0.2) | 0(0.0) | 0(0.0) | 0(0.0) | 4(0.4) | 0.06 |
| Antihypertensive medications | 667(24.5) | 138(14.6) | 109(26.2) | 90(21.7) | 330(35.0) | <0.01 |
| BMI (km/m^2^) | 23.7±2.0 | 22.0±2.4 | 24.6±3.0 | 23.2±2.4 | 25.1±2.8 | <0.01 |
| FPG (mmol/L) | 5.7±1.0 | 5.3±0.5 | 5.64±0.5 | 5.6±0.6 | 6.1±1.4 | <0.01 |
| Fasting insulin (mmol/L) | 6.2(4.4-8.8) | 4.2(3.2-5.3) | 8.6(7.3-10.6) | 4.9(4.0-5.5) | 9.1(7.5-11.6) | <0.01 |
| TG (mg/ dL) | 1.7±1.2 | 1.0±0.3 | 1.1±0.3 | 2.3±1.3 | 2.5±1.3 | <0.01 |
| eGFR (mL/min/1.73 m^2^) | 102.3±11.7 | 102.9±11.3 | 102.1±12.1 | 102.1±11.6 | 101.9±12.1 | 0.56 |
| LDL (mmol/L) | 2.8±0.8 | 2.7±0.7 | 2.8±0.7 | 2.9±0.9 | 2.9±0.8 | <0.01 |

HOMA-IR, homeostasis model assessment insulin resistance; TyG, the triglyceride glucose index; TIA, transient cerebral ischemia; BMI, body mass index; FPG, fasting plasma glucose; TG, Triglyceride; eGFR, estimated glomerular filtration rate; SD, standard deviation.

The TyG index and HOMA-IR were divided into high and low groups by using median as cut-off value. Median TyG: 8.76; median HOMA-IR: 1.55.

**Table S3. Prediction performance of the TyG index and HOMA-IR.**

| Insulin resistance | AUC (95%CI) | P value | IDI | P value | NRI (%) | P value |
| --- | --- | --- | --- | --- | --- | --- |
| Presence of intracranial plaque |  |  |  |  |  |  |
| Basic model + HOMA-IR | 0.653(0.625, 0.682) | Reference | Reference |  | Reference |  |
| Basic model + TyG | 0.657(0.628, 0.686) | 0.42 | 0.003(0.001, 0.006) | <0.01 | 18.01(7.75, 28.27) | <0.01 |
| Presence of extracranial plaque |  |  |  |  |  |  |
| Basic model + HOMA-IR | 0.607(0.585, 0.630) | Reference | Reference |  | Reference |  |
| Basic model + TyG | 0.609(0.587, 0.631) | 0.54 | 0.002(0.0004, 0.004) | 0.02 | 10.32(2.51, 18.14) | <0.01 |
| Presence of coronary plaque |  |  |  |  |  |  |
| Basic model + HOMA-IR | 0.707(0.687, 0.726) | Reference | Reference |  | Reference |  |
| Basic model + TyG | 0.707(0.688, 0.727) | 0.82 | 0.001(-0.001, 0.003) | 0.27 | 9.57(1.98, 17.16) | 0.01 |

HOMA-IR, homeostasis model assessment insulin resistance; TyG, the triglyceride glucose index; AUC, area under the curve; NRI, net reclassification improvement; IDI, integrated discrimination improvement.

Basic model included age, sex, current smoking, current drinking, BMI, glomerular filtration rate, low-density lipoprotein levels, medical history of TIA, stroke, coronary disease, hypertension and dyslipidemia, using of antihypertensive and antiplatelet medications
